# Lung Herniation as a Result of Cardiopulmonary Resuscitation (CPR): A Case Report and Literature Review

**DOI:** 10.7759/cureus.37262

**Published:** 2023-04-07

**Authors:** Pia Fuller, Ibrahim Almafreji, Stephen Cole

**Affiliations:** 1 Emergency Medicine, NHS Tayside, Ninewells Hospital, Dundee, GBR; 2 Intensive Care Unit, NHS Tayside, Ninewells Hospital, Dundee, GBR

**Keywords:** cpr, intensive care medicine, cardio thoracic surgery, in hospital cardiac arrest, cardiopulmonary resucitation, pulmonary herniation, lung herniation

## Abstract

Lung herniation is a rare complication following cardiopulmonary resuscitation (CPR) and is defined as a protrusion of lung parenchyma through the thoracic wall. This article presents a case in which a patient presented to the hospital with sepsis secondary to community-acquired pneumonia. A 74-year-old female with a background of chronic obstructive pulmonary disease (COPD) suffered a sudden pulseless electrical activity (PEA) cardiac arrest while being managed in the acute medical ward. The CT following the return of spontaneous circulation (ROSC) demonstrated multiple bilateral anterior rib fractures and herniation of the right lung through the right lateral thoracic wall. She was managed in the ICU with ventilatory and cardiovascular support for four days until she suffered a second cardiac arrest, where resuscitation was unsuccessful. In addition to this case report, a literature review was carried out, given the rarity of this pathology. The literature provides only 13 articles on lung herniation due to CPR. The most common injury pattern was anterior rib fractures leading to anterior lung herniation. In our case report, the herniation was away from the fracture site at the lateral chest wall. A common complication was surgical emphysema in several of the articles, as was in our case. The surgical intervention appears to be indicated in large hernias, incarceration, or those causing pain and respiratory compromise. In our case, conservative management was elected, given the patient’s significant persistent cardiovascular instability unsuitable for interhospital transfer. A high index of suspicion should be adopted for patients who undergo a prolonged period of CPR, including frail patients with underlying health conditions such as chronic lung disease.

## Introduction

Lung herniation is a rare complication of cardiopulmonary resuscitation (CPR). It is defined as a protrusion of lung parenchyma through the thoracic wall and is classified according to location and etiology [1,2]. It is approximated that 80% of thoracic wall lung herniation is most commonly related to trauma either blunt or penetrating and after chest wall surgery [1]. This case report demonstrates the rare occurrence of lung herniation following CPR.

## Case presentation

A 74-year-old female presented to the acute medical unit (AMU) with 24 hours of worsening dyspnea and productive cough. She was an ex-smoker with a 40-50 years smoking history and chronic obstructive pulmonary disease (COPD). She suffered a deterioration in her respiratory function over the preceding two years, requiring triple therapy inhalers (inhaled corticosteroids [IHS], long-acting beta-agonists [LAMA], long-acting muscarinic antagonists [LAMA]) and having received multiple acute courses of antibiotics and steroids in the community for infective exacerbations of COPD (IECOPD). She was reportedly independent in the community, and her past medical history included osteoporosis, a known aortic aneurysm, non-alcoholic fatty liver disease (NAFLD), and gallstones. Physical examination revealed a new oxygen requirement with oxygen saturations of 82% on room air, tachycardia, and pyrexia with stable blood pressure. Initial management for IECOPD was commenced and escalated to full sepsis protocol due to ongoing deterioration in the form of increasing oxygen requirements and persistent tachycardia. After 10 hours into her admission, she suddenly collapsed into PEA cardiac arrest. Immediate CPR following advanced life support (ALS) protocol was commenced. With high-quality manual CPR administered by trained staff throughout and 4x adrenaline (10ml of 1:10000 IV) given, return of spontaneous circulation (ROSC) was achieved after 29 minutes of arrest time. Bedside echocardiogram and chest X-ray at this time were unremarkable. Arterial blood gas demonstrated a metabolic acidosis with type 2 respiratory failure. 

**Figure 1:**
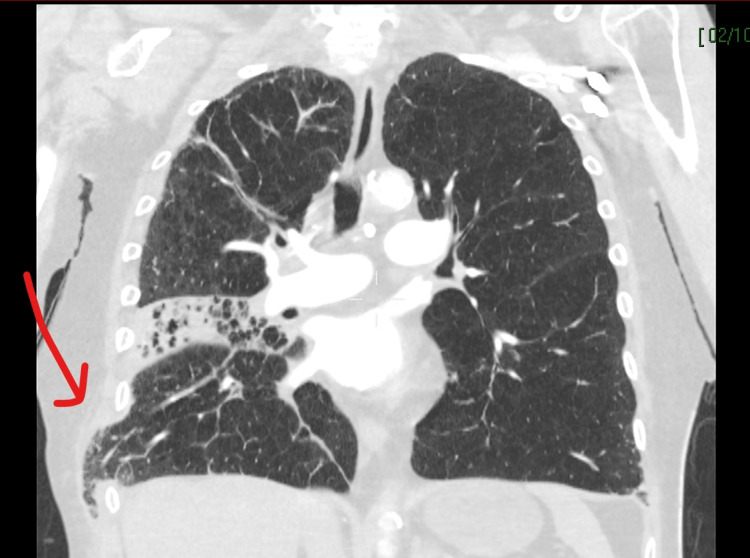
CT chest (coronal view) demonstrating right lateral lung herniation below the eighth rib and middle lobar consolidation. Red arrow pointing to lung herniation.

**Figure 2:**
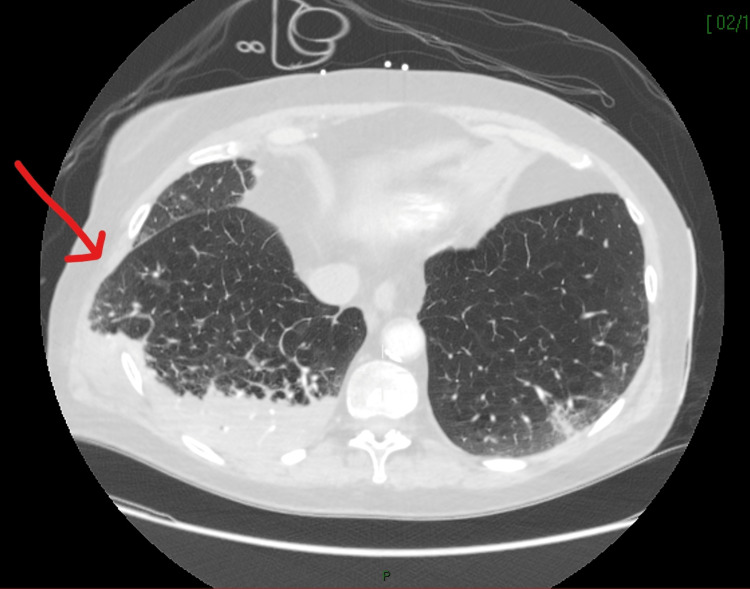
CT chest (axial view) demonstrating right lateral lung herniation and right lower lobe consolidation. Red arrow pointing to lung herniation.

She was transferred to the ICU for standard post-cardiac arrest care, including intubation, ventilation, sedation, and required inotropes for cardiovascular support. Owing to the sudden collapse, she underwent diagnostic imaging, including CT of the head, chest, and abdomen, to rule out pulmonary embolism and intracranial hemorrhage as a cause for cardiac arrest and establish the source of sepsis. This demonstrated right lower, middle, and upper lobe consolidation with bi-basal atelectasis, a likely source of sepsis. Multiple bilateral anterior rib fractures, in keeping with CPR, and herniation of the right lung through the right lateral thoracic wall were noted (Figures 1-2). High-resolution CT performed one month before admission was available for comparison and showed continued extensive emphysematous change, as noted on both CT reports.

She required a ventilator and cardiovascular support throughout her ICU admission and remained sedated with propofol and alfentanil. Significant chest wall bruising to the anterior and right lateral chest was noted, with a small palpable mass and some surgical emphysema at the right lateral wall over the site of the herniation. While primary rib fixation was discussed, her pivotal problem was persistent cardiovascular instability, and she remained too clinically unstable for safe interhospital transfer for surgical intervention. Neurology remained poor despite weaning sedation. There was no requirement for renal replacement therapy. Broad-spectrum antibiotics were continued for sepsis. She suffered a second cardiac arrest four days into her ICU admission due to persistent cardiovascular instability and multi-organ failure. Despite CPR and maximal medical support, this was a terminal event.

## Discussion

Literature review

Lung herniation following CPR is a rare event. Using the search terms ‘cardiopulmonary resuscitation,’ ‘CPR,’ ‘lung herniation,’ and‘ pulmonary herniation’ across Ovid, EMBASE, and PubMed search engines, 13 articles and case studies have been recorded prior to this report. Detailed results of the literature review are presented in Table 1. Of the 13 articles, one was unable to distinguish between surgical emphysema and lung herniation and was therefore removed for the purposes of analysis [3]. A total of 10/12 patients were 65 years or over, with seven male (1x gender undocumented) and three with known COPD. Only three diagnoses of lung herniation were on the day of cardiac arrest, with some prolonged to over one week. The most common injury pattern was anterior rib fractures leading to anterior lung herniation. One case documented herniation without fracture, and in our case report, the lung herniation was away from the fracture site at the lateral chest wall. The cohort had variable outcomes, with four receiving cardiothoracic surgery and others for chest drain insertion and conservative management.

**Table 1:** Literature review results. PMHx: Past medical history; Dx: Diagnosis; LH: Lung herniation; ESRF: End-stage renal failure; CXR: Chest X-ray; D1: Day 1; U/K: Unknown; OOHCA: Out of hospital cardiac arrest; AMI: Acute myocardial infarction; VF: Ventricular fibrillation; BIPAP: Bi-level positive airway pressure ventilation; F/U: Follow-up; SOB: Shortness of breath.

Case / Author	Age	Sex	Cause of cardiac arrest	PMHx:	Method of CPR (manual vs mechanical)	Duration of CPR	Day to Dx of LH	Modality of Dx	Injury Pattern – CPR related	Notes	Outcome
Lung herniation: a rare result of CPR/ Nawrocki J et al. [4]	70	M	ESRF on dialysis: hemorrhagic shock from fistula site		Manual	Unknown	0	Unknown	- Multiple consecutive right anterior rib and costochondral junction fractures - Flail chest - Anterior herniation of the lung with an associated moderate hemothorax	Right anterior rib and costochondral junction fractures resulting in flail chest and a large anterior herniation of the lung with an associated moderate hemothorax	Surgery offered – conservative measures chosen
Lung herniation after cardiopulmonary resuscitation/ Dugan JP et al. [5]	70	M	Presented with lobar pneumonia	COPD	Manual	10 minutes	1	CT post thoracostomy for diffuse surgical emphysema	- Dissociation between the sternum and right anterior ribs with cartilaginous separation - Comminuted and severely displaced rib fractures - Bronchopleural fistula - Lung herniation	Inter-operative – right lung herniation at site of fractures	Thoracoscopic surgery with right upper lobe wedge resection, right rib 4-7 fixation
Lung herniation after CPR/Ferreira M et al. [6]	81	u/k	Angioedema secondary to lisinopril		Manual	Unknown	4	CXR then CT on D5, two days after extubation. Surgical emphysema	- Subcutaneous emphysema, - Anterior herniation of the left lung through 5 fractured ribs - Bilateral air dissection of the muscular plans	Anterior herniation of left lung through rib fractures	Thoracic surgery with hernia reduction
Lung herniation after CPR/Batra AK [7]	65	F	Collapse as psychiatric inpatient	Nil	Manual	Unknown	4 hours	CXR	- Sternal fracture - Rib fracture - Costal cartilage separation	Soft collapsible swelling anteriorly at sternal angle that expands and collapses with ventilator. No CT done!	Conservative management. Patient died within a couple of weeks.
Lung herniation after cardiopulmonary resuscitation with mechanical chest compression device: first case report and literature review/Chan HY et al. [8]	86	F	COPD exacerbation and hypercapnia	COPD	Mechanical	60 minutes	9	CT Surgical emphysema D5 with progression	- Herniation of the left upper lobe via the second and third intercostal spaces - Fractures of the left second to fifth lateral ribs, right fourth to fifth anterior ribs, and sternal body - Left pneumothorax - Anterior hemomediastinum - Subcutaneous emphysema	Herniation of the left upper lobe via the second and third intercostal spaces; fractures of the left second to fifth lateral ribs, right fourth to fifth anterior ribs, and sternal body	Chest tube was inserted, and subsequent wedge resection of the left upper lobe and pneumolysis were performed
Intercostal lung herniation after cardiopulmonary resuscitation masked by diffuse subcutaneous emphysema/Lee HT and Wang AY [9]	65	M	OOHCA secondary to AMI		Manual and mechanical	Unknown	1	Surgical emphysema D1 CXR and CT Dx with serial imaging following pig-tail chest drain insertion	- Subcutaneous emphysema	Drainage and then discharged D25	CT is the preferred modality for diagnosing lung hernias
Lung herniation post cardiopulmonary resuscitation/Aggarwal S, Loehrke M [10]	56	M	VF cardiac arrest – unclear cause		Manual	10 minutes	7	CT Unable to tolerate BIPA post extubation	- Displaced fractures from the third to the sixth ribs - Herniated lung right-sided upper and middle lobe	CT demonstrated: displaced fractures from the third to the sixth ribs and a herniated right-sided upper and middle lobe of the lung tissue	Surgical repair of a large right lung hernia with a right pectoralis major muscle flap, open-reduction and internal fixation of multiple right-sided rib fractures
Incidental finding of lung herniation after cardiopulmonary resuscitation/Talebi S et al. [11]	85	F	Cardiac arrest 1 year before case reported		Manual	Unknown	At time of event and reported 1 year later	CT	- Herniation of the lung into the left chest wall soft tissue at the level of seventh intercostal space	Herniation of the lung into the left chest wall soft tissue at the level of seventh intercostal space	Lung herniation without rib fracture. No surgical intervention
Lung hernia associated with hemothorax following cardiopulmonary resuscitation/ Emberger JS Jr et al. [12]	69	M	Cause unclear	COPD	Manual	Unknown	2	CT	- Fractures of the anterior right third, fourth, and fifth ribs, and the anterior left fourth and fifth ribs	Large left pleural effusion was noted on the morning chest radiograph> f/u CT revealed herniation of lung through the 4th rib fracture, and a large hemothorax of the left lung	Chest tube insertion No other intervention Spontaneous resolution
A rare complication of cardiopulmonary resuscitation/ Kottachchi DT et al. [13]	74	M	Massive oesophageal variceal haemorrhage		Manual	2.5 minutes	7	CT	- 2 left rib fractures - Herniation of the lung parenchyma through one of the fractures - Left-sided pneumothorax	2 left rib fractures and herniation of the lung parenchyma through one of the fractures	Chest tube insertion and conservative management. Resolution (CT proven) two weeks post incident
Lung herniation after cardiopulmonary resuscitation/ Sprague LD and Ferrigni FJ [14]	63	M	Arrest at work. Pulmonary embolus	Unknown	Manual	5 mins	1	CT	Rib fracture from CPR Lung herniation anteriorly at the site of rib fracture Subcutaneous emphysema, pneumopericardium and pneumomediastinum No pneumothorax	- Patient developed SOB and subcutaneous emphysema after extubation and needed to be re-intubated prior to undergoing CT. - Patient was hemodynamically stable after ROSC. Thus, PE demonstrated on CT was treated with IV heparin. No thrombolysis.	Wedge resection of the left lung. Tube thoracotomy. Long hospital stay, survived, and made a full neurological recovery.
Lung herniation or subcutaneous emphysema after cardiopulmonary resuscitation?/ Oncel M et al. [3]	73	F	Cardiac arrest at home – unclear cause	Liver cirrhosis	Manual	>5 minutes	0	CT	Subcutaneous emphysema – chest wall during CPR CT showed left anterior lung herniation	Subcut emphysema initial clinical sign followed by CT evidence of herniation.	Chest drain inserted. No surgery. Unknown patient outcome.

Multiple rib fractures and sternal fractures are common resulting features after CPR, with up to 97% and 43% of people affected, respectively [15]. Despite the high prevalence of thoracic wall injury, lung herniation remains a rare incidence following CPR, with previous studies demonstrating no evidence in a cohort of over 700 individuals [16]. Frailty and preexisting medical conditions can predispose an individual to a more extensive injury pattern. This, combined with high-quality chest compressions, puts individuals at higher risk of severe thoracic injury after CPR [8]. With the introduction of mechanical chest compression devices, two case reports in the last five years demonstrate a resultant lung herniation. However, further evidence is needed to ascertain if this will increase the incidence of thoracic trauma compared to manual chest compression [8,9]. CT has been the modality to identify lung herniation throughout all reported cases. Physical examination may identify a bulge at the site of the lung herniation, and clinically there has been increased respiratory distress both in ventilated and non-ventilated patients. Chest X-ray (CXR) commonly reveals rib fractures, surgical emphysema, and loculated air pockets but is not the imaging modality of choice for lung herniation [7,13,17]. Surgical emphysema on CXR has been shown to mask underlying pathology and therefore has been the main indication for a CT in these cases [5,9]. In each case, CT has demonstrated the site and extent of the lung herniation and other injury patterns [17]. The lung herniates most commonly anteriorly or at the fracture site [4,6,14]. In our case, the lung herniated laterally away from the fracture site. Herniation may occur even in the absence of rib fractures [11]. Four patients underwent cardiothoracic surgery for rib fixation and hernia reduction [5,6,10,16]. Surgical intervention is indicated in large lung hernias, those that are incarcerated, irreducible, or causing pain and respiratory compromise [6]. Most patients were for conservative management, including chest drain insertion; however, this was in the context of haemothorax and pneumothorax. Some cases of those under conservative management were shown to resolve spontaneously on serial CTs [12,13].

## Conclusions

Lung herniation remains a rare complication of CPR. A high index of clinical suspicion should be present in those who underwent an extended period of CPR and in the frail with underlying health conditions. CT is the best modality for diagnosing lung herniation and has been shown to have the highest detection rate for CPR-related injuries. Given the emphasis on high-quality CPR and the use of mechanical chest compression devices in an aging population, clinicians need to be aware of this rare event, as timely recognition and management may improve survival.
